# Transcatheter Aortic Valve Implantation: A Report on Serbia's First Systematic Program

**DOI:** 10.3389/fcvm.2022.882854

**Published:** 2022-05-24

**Authors:** Darko Boljevic, Milovan Bojic, Mihajlo Farkic, Dragan Sagic, Dragan Topic, Vladimir Kovacevic, Jovana Lakcevic, Stefan Veljkovic, Milan Dobric, Sasa Hinic, Nenad Ilijevski, Marko Nikolic, Aleksandra Kaludjerovic, Matjaz Bunc, Aleksandra Nikolic

**Affiliations:** ^1^Dedinje Cardiovascular Institute, Belgrade, Serbia; ^2^School of Medicine, University of Banja Luka, Banja Luka, Bosnia and Herzegovina; ^3^School of Medicine, University of Belgrade, Belgrade, Serbia; ^4^University Clinical Center Ljubljana, University of Ljubljana, Ljubljana, Slovenia

**Keywords:** TAVI, aortic valve, aortic stenosis, TAVI in Serbia, structural heart disease interventions

## Abstract

**Introduction:**

Severe aortic stenosis, a highly-common valve disease in the elderly, has a poor prognosis if left untreated. To address the concern of effective procedures for severe aortic stenosis, a systematic TAVI program was established at the Dedinje Cardiovascular Institute (Belgrade, Serbia).

**Methods:**

Our cohort was composed of 56 patients (74±15 years old). The mean logistic EuroScore was 10.17%; the mean Society of Thoracic Surgeons score was 3.22%. One third of the patients were categorized as class III or IV of the New York Heart Association (NYHA). The valves selected for use were either self-expandable or balloon expandable (Evolut R, Medtronic; Acurate Neo, Boston Scientific and Myval, Meril). The choice of valve type was made by the Institute's Structural Heart Team, in accordance with the patient's native aortic valve, size and calcification of ilio-femoral vessels, as well as the need for alternative access. TAVI procedure was conducted according to current guidelines provided by the European Society of Cardiology.

**Results:**

The procedure success rate was 100%. Trans-femoral approach was achieved in 100% of patients; percutaneously in 87.5%, while a surgical cut was necessary in 12.5%. No patient showed moderate or severe aortic regurgitation after the procedure, although trace or mild regurgitation was recorded in 30.3%. Permanent pacemaker was implanted in one patient (1.78%), contrast induced acute kidney injury occured in one patient (1.78%), no stroke was recorded, and three pseudo-aneurysms which required surgical intervention occurred. Three patients required blood transfusions (5.33%). A 30-day all-cause mortality rate was 1.78%.

**Conclusion:**

The Dedinje Cardiovascular Institute spearheaded all efforts to establish a TAVI program in Serbia. Our initial TAVI results are promising, encouraging, and comparable with the results of previous large randomized trials. This initial experience opens the door for further development with a goal of our Institute to become a high-volume TAVI center.

## Introduction

Severe aortic stenosis, a highly-common valve disease in the elderly, has a poor prognosis if left untreated (1). Although the prevalence of aortic stenosis is on the rise due to prolonged lifespan and the aging of the population in developed countries (2), the treatment of severe aortic stenosis had been limited to open heart surgery prior to the advent of TAVI as a novel less-invasive procedure. Albeit that open-heart surgery may still be exclusively practiced in those areas in which TAVI has not been introduced, TAVI is comparable to such procedures in effectiveness of treatment and health outcomes, as well as improving the immediate post-procedural quality of life of the patient, and reducing hospital stay and overall costs. TAVI procedures, moreover, pose less risk for complications, as evidenced by their reliable survival rate (3). Even though TAVI poses less of a risk to the patient and their health (4), care must be taken when overestimating its use as even minor health concerns may lead to unfavorable outcomes (5). Recent European Society of Cardiology Guidelines recommend trans-femoral TAVI as the preferred mode of intervention in patients ≥75 years of age, as well as for specific patient groups <75 (6).

There are few published reports regarding the introduction of systematic TAVI program in different countries/regions. Their findings support implementation of TAVI procedures in different risk-groups, including patients with high, medium and low risk. From 2007–2008, Spargias et al. carried out research in high-risk patients who had been classified at too high risk to undergo open heart surgery. Of their 12 patients who would otherwise not be able to undergo any surgery, all TAVI procedures were successful. They extrapolated that these findings could serve as an example of the extent to which a wider TAVI program could be applied throughout Greece in treating high risk cardiovascular patients (7). Parma et al. analyzed data coming from Poland; they found that the dispersal of a TAVI program throughout Poland would better suit those awaiting open-heart procedures, which was hindered in its growth, reaching only 5.12% of the total potential patient population. Moreover, they asserted that TAVI could be better applied to medium or lower risk patients (8).

In order to introduce TAVI procedures, and to address the growing need for effective treatment options for severe aortic stenosis, a systematic TAVI program was established at the Dedinje Cardiovascular Institute (Belgrade, Serbia). This paper presents our results over a three-year time frame with 56 treated patients. As the Serbian healthcare system operates as a state-run enterprise with institutions that receive national funding to carry out their services (9, 10), no TAVI program could be set up prior to receiving medical approval from the National Health Fund of the Republic of Serbia. To avoid this timely and costly process, our program was set up as a commercial enterprise in which patients were required to pay out of pocket for the TAVI procedure. From April 2019 to December 2021, patients who were diagnosed with severe aortic stenosis at the Dedinje Cardiovascular Institute or any other referring hospital within the Republic of Serbia, were advised that they may undergo this procedure if they could provide funding. All patients had to meet the criteria of the European Society of Cardiology guidelines (i.e. they possessed a high STS and EuroScore or other contraindications for open heart surgery such as the presence of porcelain aorta, severe lung disease, or chest radiation or deformity). There were no age criteria, meeting the standard set out before August 2021 (11). The presence of the TAVI program as a commercial program run within a state institution provided impetus for the National Health Fund of the Republic of Serbia to eventually approve its public funding. Apart from teaching purposes for the participating medical professionals, the program also served as an initial evaluation of the effectiveness of TAVI procedures in the local population.

## Methods

### Study Population

Our cohort consists of 56 consecutive patients who underwent TAVI procedure from April, 2019 to December, 2021 at the Dedinje Cardiovascular Institute (Belgrade, Serbia). The population comprised of 29 (51.7%) male, and 27 (48.3%) female, who were 74 ± 15 years old (range 62 – 89). Although inordinately younger than in other studies, all patients who were under the age of 75 were included if they presented with a porcelain aorta or any other comorbidity, such as chest wall deformity, chest irradiation or pulmonary fibrosis, that could increase surgical risk. Most of the patients who were at low- or intermediate-risk were initially offered to undergo open-heart surgery, but the patient refused to due to inconvenience. As they had the funds to do so, the patients opted to have the less invasive procedure (TAVI) which resulted in the population's predominantly low-risk profile. The remaining patients were deemed to be at too high risk or completely inoperable for open-heart surgery. According to criteria set out for the EuroScore and STS, patients were categorized into the following risk groups: low - 32 patients (57%), medium - 14 patients (25%), and high - 10 patients (17%). All patients were able to pay for the procedure according to the official pricelist. All cases were presented to the structural heart team of our Institute in order to assess the feasibility and safety of undergoing the TAVI procedure.

All TAVI patients of the study were entered into the TAVI registry of the Institute which also recorded their baseline characteristics, procedural details, device used and clinical outcomes. They attended regular follow-ups in the Institute's outpatient clinic as well as regular echocardiographic follow-ups whose results were included into the database. Written informed consent was obtained before the procedure, as well as consent for data processing.

### Patient Selection and Pre-TAVI Assessment

Pre-interventional patient work-up included transthoracic echocardiography in order to confirm the diagnosis (i.e, to assess to AVA and mean gradient, and function of left and right ventricles), a multislice computed tomography to assess aorta and aortic valve dimensions and morphology, grade and distribution of the calcifications, annulus dimension and ilio-femoral tract. A coronary angiogram was performed to evaluate coronary arteries, Doppler ultrasound for carotid arteries, and standard laboratory workup. If required, percutaneous coronary intervention (PCI) was performed prior to TAVI. As no guidelines were available to determine when a PCI should be performed post or prior to the TAVI, all proximal lesions were treated while all others were left to the operator to decide the best course of action. This was procedurally done in accordance with the protocols of the Dedinje Cardiovascular Institute of Belgrade, Serbia, where the TAVI's were implanted.

### Valve Prosthesis

**Figure 1:**
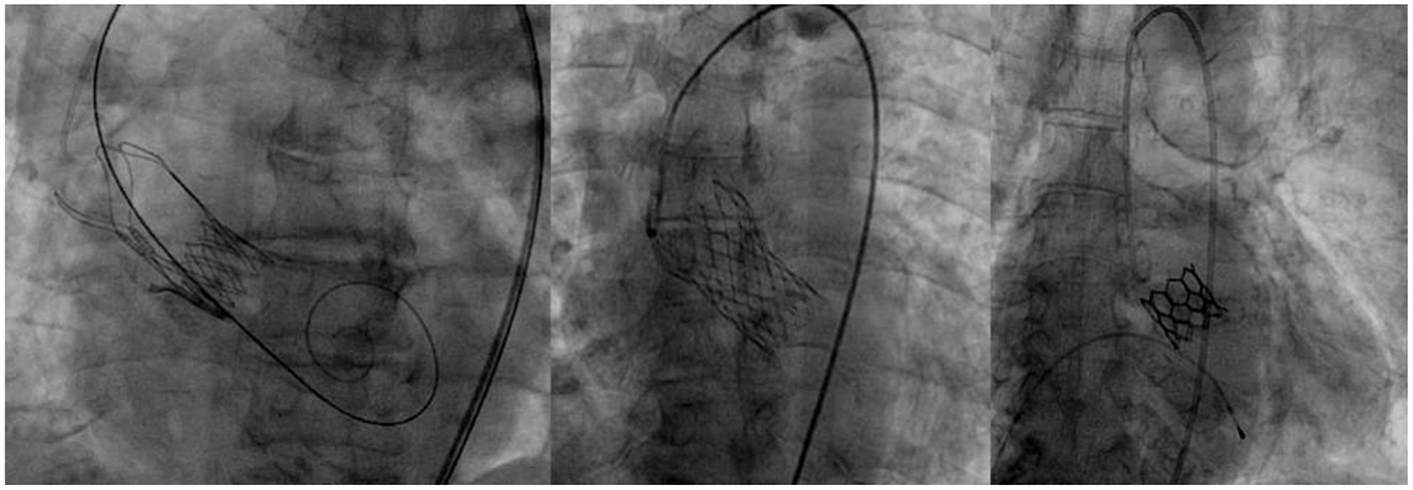
Acurate Neo, Boston Scientific; Evolut R, Medtronic; and Myval, Meril.

Valves were selected according to each patient's characteristics among the following self-expandable (*Evolut R, Medtronic and Acurate Neo, Boston Scientific*) or balloon expandable ones (*Myval, Meril*) (Figure 1). These three devices were used for starting the program, because the TAVI program was operating during the peak of the Covid pandemic, when available valves from one manufacturer were limited. It was also agreed that it would be best to allow a sort of variety of devices in order to match different patient profiles. The choice of valve type was made by the Institute's Structural Heart Team, in accordance with the patient's native aortic valve, size and calcification of ilio-femoral vessels as well as the need for alternative access. Criteria to use either the self-expandable or balloon type was based on criteria set by the patient's health and cardiovascular history. Balloon expandable valves were used in those who had severe calcifications which might lead to valve leakage, as well as in those showing a high risk for permanent pacemaker implantation. In addition, in those patients who presented with severe coronary artery disease that might lead to PCI in the future, balloon type valve was the preferred choice. Due to their supra-annular leaflets, self-expandable valves were used in patients who had small annuli to ensure optimal hemodynamics after implantation. Moreover, in those who were at high-risk for annular rupture, self-expandable valve was used. In cases where there were no limitations set by the patient's characteristics, it was left to the individual operator to use their best judgment to choose the most appropriate valve.

### Antiplatelet and Anticoagulation Therapy

Prior to the ESC guidelines being published in August 2021, patients had been treated with lifelong Aspirin (100 mg) and Clopidogrel (75 mg) for 3 months post-implantation. Under the new guidelines, only lifelong Aspirin (100 mg) was prescribed.

An anticoagulation regimen was followed three-months post implantation in those patients who required anti-coagulation therapy, Warfarin was prescribed for the first 3 months (achieving INR 2–3). Following these 3 months, the patient was consulted, either remaining on Warfarin or changed to NOACs (preferably Apixaban 5 mg twice a day).

### Statistical Analysis

All continuous variables were expressed as mean ± standard deviation, and categorical variables as frequencies and percentages. The analysis did not incorporate any missing data imputation procedures.

## Results

### Baseline Characteristics

**Figure 2:**
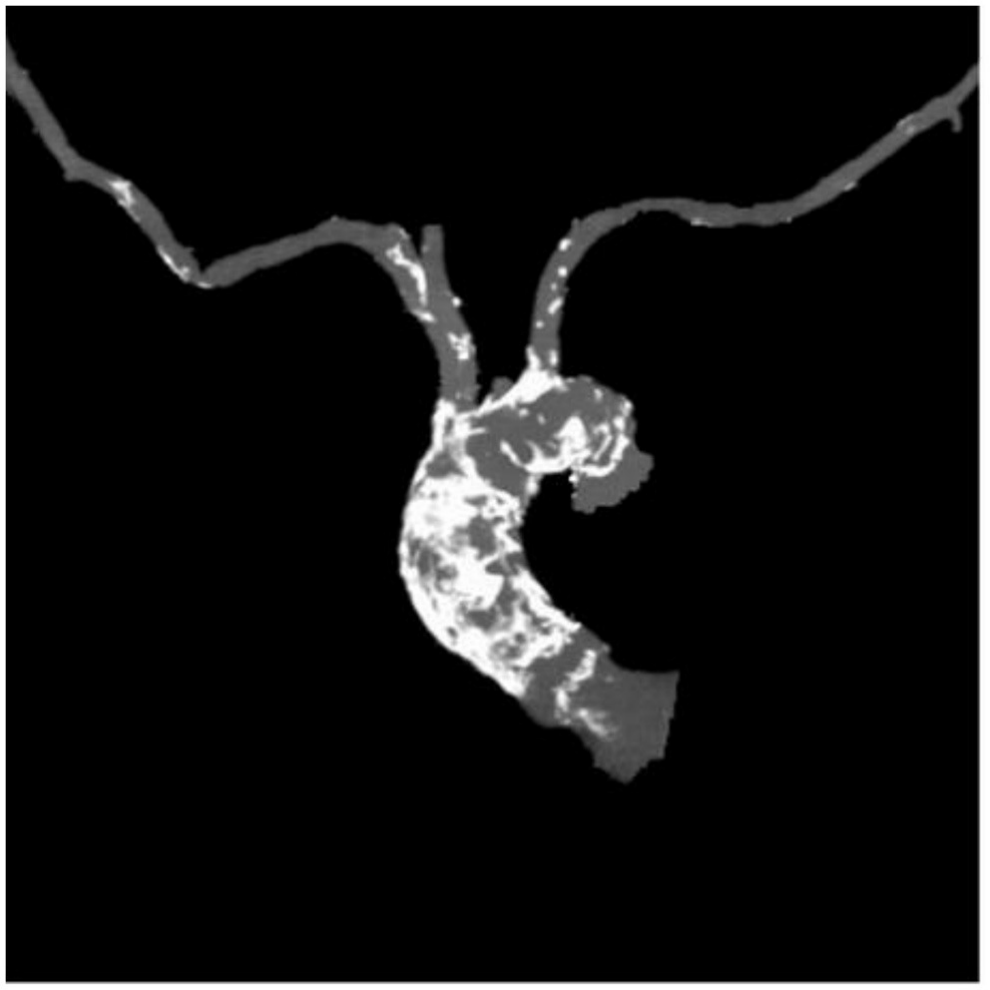
Porcelain aorta.

Table 1 outlines all baseline characteristics for the 56 patients. The patients were 29 (51.7%) male and 27 (48.3%) female, who were 74±15 years old (range 62 −89). The mean logistic EuroSCORE was 10.17%, the mean Society of Thoracic Surgeons score was 3.22%. One third of the patients were categorized as class III or IV of the New York Heart Association (NYHA). Almost half of the patients had concomitant coronary artery disease (48.2%). Myocardial revascularization was previously performed in 46.3% (PCI in 16%, CABG in 30.3%). 7.1% of patients had carotid disease, while 14.2% of patients had lower extremity artery disease. As such, 12% of patients needed to undergo a surgical cut as a vascular approach due to the nature of the disease (mainly heavy classifications). One patient also had severe stenosis of the popliteal artery. Lung disease was found in 25%, chronic kidney disease was present in 14.2% patients (i.e., one patient was on hemodialysis). The presence of porcelain aorta was considered to be direct indication for a TAVI in 14.2% of patients (Figure 2). TAVI was suggested to 2 patients due to chest deformation and previous chest irradiation. The mean left ventricle ejection fraction was 44% (range 15–65%), while the mean of the aortic pressure gradient was 45 mmHg (range 29–106 mmHg). The average aortic valve area was 0.58 cm^2^ (range 0.4–0.9 cm^2^).

**Table 1:** Baseline characteristics.

*n*	56
Age	74 ± 15
Male sex	29 (51.7%)
STS score	3.22%
Logistic EuroScore	10.17%
**NYHA class**	
NYHA II	38 (67.8%)
NYHA III or IV	18 (32.1%)
Coronary artery disease	27 (48.2%)
Previous myocardial infarction	9 (16%)
Previous cardiac intervention	17 (30.3%)
PCI	9 (16.1%)
CABG	8 (14.2%)
BAV	1 (1.78%)
Carotid artery disease	4 (7.1%)
Peripheral artery disease	8 (14.2%)
COPD or pulmonary fibrosis	14 (25%)
Oxygen-dependent	0
Creatinine more than 2 mg/dl	7 (12.5%)
Atrial fibrillation	11 (19.6%)
Permanent pacemaker	6 (10.7%)
Pulmonary hypertension	13 (23.2%)
Extensive aortic calcification	8 (14.2%)
Deleterious effects or chest irradiation	3 (5.3%)
Chest wall deformity	1 (1.78%)
Liver disease	1 (1.78%)
Aortic valve area, cm2	0.58 ± 0.22
Mean aortic gradient, mmHg	45 ± 61
Mean LVEF, %	44 ± 29
Moderate of severe mitral regurgitation	17 (30.3%)

### Procedural Outcomes

Table 2 summarizes the procedural outcomes. Procedure success was 100%. The majority of procedures were done under local anesthesia and/or conscious sedation, unless a surgical cut was used to access the common femoral artery when general anesthesia was required. A trans-femoral approach was achieved in 100% of patients (percutaneous - 87.5%, surgical cut - 12.5%). An Evolut R prosthesis was most commonly implanted. No patient showed moderate or severe aortic regurgitation, although trace or mild regurgitation was recorded in 30.3%. Post dilatation of the valve was performed in 8 patients (14.2%) who experienced at least a moderate paravalvular leak. Otherwise, there was no need for post-dilatation because of the significant transvalvular gradient. Following the procedure, both the peak and mean gradient was measured in all patients. The average peak gradient was 15 mmHg while the average mean gradient was 8 mmHg.

**Table 2:** Procedural outcome.

*n*	56
Procedure success	56/56 (100%)
Transfemoral	56 (100%)
Percutaneous	49 (87.5%)
**Protesis size**	
Evolute R, Medtronic	34 (60%)
26	5 (8.9%)
29	25 (44%)
34	4 (7.1%)
Acurate Neo, Boston Scientific	9 (16%)
S	1 (1.78%)
M	3 (5.3%)
L	4 (7.1%)
Acurat Neo 2 M	1 (1.78%)
MyVal, Meril	13 (23.2%)
21.5	1
23	3
24.5	2
26	2
27.5	3
30.5	1
32	1
Valve post-dilatation	8 (14.2%)
Second valve required	0
**Post-procedural aortic regurgitation**	
Trivial or mild	17 (30.3)
Moderate or severe	0
Open heart surgery (tamponade)	1 (1.78%)
Annular rupture	0
Coronary obstruction	0
Peak gradient	15 mmHg
Mean gradient	8 mmHg

### Adverse Events

Safety endpoints and clinical outcomes are shown in Table 3. The conversion to open heart surgery after the procedure was necessary in one patient due to a pericardial tamponade caused by right ventricle perforation due to a rapid pacemaker. New conduction abnormalities (a complete AV block) which required the implantation of a permanent pacemaker occurred in one patient (1.78%). One patient (1.78%) also died 2 weeks following procedure due to respiratory failure from severe pulmonary fibrosis. Contrast induced acute kidney injury was also registered in one patient. There was no stroke recorded, but three pseudo-aneurysms occurred, which required surgical intervention and 1 iliac artery dissection which required no intervention. In four patients, major bleeding was observed, three of whom required blood transfusions. There was gastrointestinal bleeding in two patients (duodenal ulcer and hemorrhoids), one experienced inguinal hematoma, and one patient had a pericardial tamponade.

**Table 3:** Clinical and safety outcome.

TAVI – 30 day outcome	*N* = 56
**Death**	
Death from any cause	1 (1.78%)
Death from cardiovascular cause	0
Re-hospitalization	3 (5.35%)
Death from any cause or re-hospitalization	4 (7.1%)
Major bleeding	4 (7.1%)
**Stroke or TIA**	
All	0
Stroke	0
Minor	0
Major	0
TIA	0
Death from any cause or Stroke	1 (1.78%)
Myocardial infarction	0
**Vascular complication**	
All	5 (8.92%)
Major	4 (7.14%)
Acute Kidney Injury	1 (1.78%)
Major bleeding	4 (7.1%)
**Cardiac re-interventions**	
BAV	0
Re-TAVI	0
SAVR	0
Endocarditis	0
New atrial fibrillation	0
New pacemaker	1 (1.78%)

## Discussion

This paper provides a detailed report on the first systematic TAVI program in Serbia, established within the Dedinje Cardiovascular Institute, one of the Serbia's leading cardiovascular facilities. The program, although included a limited number of patients [56], has been able to achieve successful results in the time it has operated (2019 to 2021). The program's results have contributed to the overall body of evidence showing that TAVI could be performed reliably in a cardiac catheterization laboratory by a team of trained physicians, in patients appropriate for the procedure.

Since the first TAVI device was approved and implanted, indication has been extended from patients considered inoperable or at extreme risk (2011), to high-risk (2012), to intermediate-risk (2016), and eventually to low-risk (2019) for surgery (12–14). While the results of the TAVI program at the Dedinje Cardiovascular Institute were achieved during the learning phase of the program, this series included more low to intermediate risk patients than those who were at high-risk. The predominance of low-risk patients was due to the nature in which patients were selected based on their preference to undergo a TAVI and their ability to fund the procedure, even though these patients were offered a surgical aortic valve replacement first. The 100% procedure success in the first 56 patients was accompanied by a low mortality rate (1.78% at 30 days), which are in line with the PARTNER trials and may be found to be similar to other small patient series. Lee et al., for instance, carried out a similar small trial study on a comparable population of 56 patients of similar age and who achieved alike outcomes (15). The trial program even achieved better results than in other trial TAVI programs within other medical systems, such as noted in Wongsikongman et al. and Albugami et al., which were carried out on a similar sample size and in the same timeframe (16, 17).

No perioperative myocardial infarction, annular rupture, aortic dissection, stroke, or need for second valve implantation occurred among the studied patients, and no acute renal failure necessitating dialysis was observed. While three patients did require blood transfusion, such treatment is comparable with data from the PARTNER trials. Notably, our 30-day stroke and transient ischemic attack rate is comparable with 1 – 4.6% in the PARTNER trials. Major vascular access complications are seen in approximately 10% of patients in the literature and are in part related to high-profile delivery systems and sheaths. In our sample, 4 patients (7.1%) had a major vascular complication: 3 pseudo-aneurysms which required surgical intervention and 1 iliac artery dissection which required no intervention. This was recognized and treated effectively by vascular surgeons and interventional radiologists. Conversion to open heart surgery happens in 0.1–1% (18, 19). In our study, one conversion occurred due to a pericardial tamponade caused by a rapid pacemaker. The conduction system location does generate a risk of conduction abnormalities including complete AV block and LBBB. One patient out of 56 (1.78%) required a new permanent pacemaker due to complete AV block, which is lower when compared to Evolut Low Risk Trial whose authors reported 17% of new pacemaker implantations following TAVI (20, 21). A major pitfall of TAVI so far has been occurrence of paravalvular leak (PVL), a common complication as patients undergoing TAVI typically who have heavy calcifications and difficult aortic root anatomy. PVL rates in TAVI implants may reach up to 60% (22). Our patients experienced no moderate or severe paravalvular leaks (0%) and 17 trace-to-mild PVL (30.3%).

Decision-making process for every patient must be individualized, as we did in the development phase of our TAVI program. Although the program was forced to use three different TAVI implants to meet limitations set by supply on the market from the Covid-19 pandemic, the value of rigorous patient selection by the multidisciplinary Structural Heart Valve team cannot be overemphasized in making this program effective. Realistic expectations relating to the procedure, including the management of potential complications, are explicated prior to the procedure itself. The EuroScore and STS scores are valuable in predicting mortality rate risks.

### Limitations

This research is limited in its scope due to its small sample size and single-center nature, but this is expected froum a study that describes introduction of a new systematic TAVI program. In addition, the use of multiple valves may slightly skew the overall outcomes as being positive for the program.

## Conclusions

Until 2019, Serbia had no TAVI program in practice due to its prohibitions of high costs. The Dedinje Cardiovascular Institute spearheaded all efforts to create a TAVI program in Serbia, whose initial TAVI results have been so promising and encouraging that we have received certification to implement a state-funded and approved TAVI program. This program in its development may serve as one example of many in which TAVI programs may develop in those areas in which they have not already been established as well as serve to illustrate the overall benefits of having such a program in place.

## Data Availability Statement

The original contributions presented in the study are included in the article/supplementary material, further inquiries can be directed to the corresponding author.

## Author Contributions

DB, MBo, MF, and DS conceptualized this manuscript and drafted the first manuscript. DT, JL, SV, VK, MN, and AK collected the data. MD, SH, NI, MBu, and AN revised final revision of the manuscript. All authors provided critical feedback and contributed to the manuscript.

## Conflict of Interest

The authors declare that the research was conducted in the absence of any commercial or financial relationships that could be construed as a potential conflict of interest.

## Publisher's Note

All claims expressed in this article are solely those of the authors and do not necessarily represent those of their affiliated organizations, or those of the publisher, the editors and the reviewers. Any product that may be evaluated in this article, or claim that may be made by its manufacturer, is not guaranteed or endorsed by the publisher.
